# A pilot study to investigate real-time digital alerting from wearable sensors in surgical patients

**DOI:** 10.1186/s40814-022-01084-2

**Published:** 2022-07-06

**Authors:** Meera Joshi, Hutan Ashrafian, Sonal Arora, Mansour Sharabiani, Kenny McAndrew, Sadia N. Khan, Graham S. Cooke, Ara Darzi

**Affiliations:** 1 Department of Surgery and Cancer, Imperial College London, 10th Floor Academic Surgical Unit, QEQM Building, St Mary’s Hospital, Praed Street, Paddington, London, W2 1NY UK; 2 Chelsea and Westminster Hospital, NHS Foundation Trust, London, UK; 3 Department of Primary Care & Public Health, School of Public Health, Imperial College London, London, UK; 4 Division of Infectious Diseases, Imperial College London, London, UK

**Keywords:** Wearable sensors, Vital signs, Continuous monitoring

## Abstract

**Background:**

Continuous vital sign monitoring may identify changes sooner than current standard monitoring.

**Objective:**

To investigate if the use of real-time digital alerts sent to healthcare staff can improve the time taken to identify unwell patients and those with sepsis.

**Design:**

A prospective cohort study design.

**Setting:**

West Middlesex University Hospital, UK.

**Participants:**

Fifty acutely unwell surgical patients admitted to hospital.

**Intervention:**

Patients wore a lightweight wearable sensor measuring heart rate (HR), respiratory rate (RR) and temperature every 2 min whilst standard intermittent ward monitoring of vital signs was performed by nurses. Digital alerts were sent to healthcare staff from the sensor to a smartphone device. All alerts were reviewed for recruited patients to identify the exact time on the sensor in which deterioration occurred. The time to acknowledgement was then reviewed for each action and an average time to acknowledgement calculated.

**Results:**

There were 50 patients recruited in the pilot study, of which there were vital sign alerts in 18 patients (36%). The total number of vital sign alerts generated in these 18 patients was 51. Of these 51 alerts, there were 7 alerts for high HR (13.7%), 33 for RR (64.7%) and 11 for temperature (21.6%). Out of the 27 acknowledged alerts, there were 2 alerts for HR, 17 for RR and 8 for temperature. The average time to staff acknowledgement of the notification for all alerts was 154 min (2.6 h).

There were some patients which had shown signs of deterioration in the cohort. The frequency of routine observation monitoring was increased in 2 cases, 3 patients were referred to a senior clinician and 2 patients were initiated on the sepsis pathway.

**Conclusion:**

This study demonstrates the evaluation of digital alerts to nurses in real time. Although not all alerts were acknowledged, deterioration on the ward observations was detected and actions were taken accordingly. Patients were started on the sepsis pathway and escalation to senior clinicians occurred. Further research is required to review why only some alerts were acknowledged and the effects of digital alerting on patient outcomes.

**Trial registration:**

ClinicalTrials.gov, NCT04638738

## Key messages regarding feasibility

i) What uncertainties regarding feasibility existed prior to this study?
  Would we be able to engineer the clinical areas so that alerts could be sent in real time? Could novel wearable sensors providing continuous vital sign measurements be used in a hospital ward setting? Would the busy clinical team acknowledge the alerts? Would digital alerts identify unwell patients? Would digital alerts identify patients with sepsis?
ii) What are the key feasibility findings from this study?
  Wearable sensors can successfully be used in clinical areas providing continuous vital sign remote monitoring. Clinical staff did acknowledge many alerts how not all alerts from the sensor were detected. However, patient deterioration using the wearable sensor technology was identified with increased monitoring, alerting to a senior clinician and patients being commenced on the sepsis pathway.
iii) What are the implications of the findings on the design of a future study?
  A large randomised control trial is required to review why only some alerts were acknowledged and the effects of digital alerting on patient outcomes.

## Introduction

Delayed detection of patient deterioration in the hospital is a major cause of morbidity and mortality and is mostly caused by human-related monitoring failures [1–3]. During patient deterioration, there are changes in a patient’s physiological parameters. An early step in recognition is changes in a patient’s vital signs [4, 5]. Vital sign changes measured as part of routine clinical care for hospitalised patients may be present several hours prior to clinical events such as cardiac arrest, death and intensive care unit admission [6]. Unfortunately, existing systems are not reliably detecting deteriorating patients quickly and 39% of acute emergency patients admitted to critical care units are referred late [2].

Reasons for delays in the detection of patient deterioration include incomplete and infrequent monitoring [7]. In a literature review, the inability to reliably detect deterioration was broadly categorised into patient variables, nursing factors and organisational factors [8]. Individualised patient factors included the relative importance of some vital signs such as respiratory rate compared to others [9, 10], although each additional abnormal vital sign is associated with an increase in mortality risk [11]. Nursing factors can lead to failures in patient deterioration and these include a lack of clinical knowledge, problems with roles and responsibilities (with vital sign monitoring often performed by non-registered nurses) and the failure of reporting or raising an alarm [8]. Organisational factors can include the observation charts themselves, with those having track and trigger systems, the use of colour coding and banding to highlight deterioration performing better [8].

Sepsis is a major cause of patient deterioration and is defined as a ‘life threatening organ dysfunction caused by a dysregulated host response to infection’ [3]. The mortality for severe sepsis is as high as one in four [12]. The cost of sepsis is colossal with total US costs of sepsis accounting for $24 billion annually [4]. A retrospective multicentre study found for every hour’s delay in the treatment of sepsis, the risk of death increases by 7.6% [5]. Currently, sepsis is identified late in most cases. The causes of the late detection of sepsis are largely unknown. It is likely to be similar to the factors described above through a combination of patient, nursing staff and organisational factors. The lack of a single biochemical test for sepsis is also likely to play a role [13]. Changes in vital sign parameters in early sepsis are often subtle and can be difficult to interpret [14]. However, it is thought that the early identification and treatment of sepsis may reverse the infection process found in sepsis [3], improve both patient outcomes and reduce avoidable deaths [15–17]. Changes in a patient’s vital signs are one of the early identifiers of sepsis, especially changes in temperature and respiratory rate as the respiratory system is the most common site of sepsis [6]. In all patients, respiratory rate is the best predictor of patient deterioration [8].

The UK NICE recommends physiological observations to be recorded every 12 h and this increased in deterioration [18]. The vital signs that are routinely measured are heart rate, respiratory rate, oxygen saturations, blood pressure and temperature. Most hospitals worldwide rely on intermittent vital sign observations for patients on general wards [19].

In deteriorating patients, the frequency of vital sign monitoring can be increased to every 20–30 min. A worsening in a patient’s condition requires increasing the physical monitoring of a patient. This may occur on a general ward or through more specialist settings such as higher dependency units or intensive care units. However, this will occur upon deterioration being recognised. In addition, deterioration between intermittent observations may be missed. The optimal sampling frequency for vital sign measurements is unknown [20].

Novel wearable sensors may identify unwell patients in a timelier manner through continuous monitoring. These new devices can measure vital signs more frequently than standard current nursing monitoring [21]. They are seen as a positive tool in detecting patient deterioration [22]. The utility of real-time alerting to the clinical team through smartphone/handheld devices is unknown.

The objective of this study was to investigate the use of continuous monitoring through wearable sensors, real-time digital alerts sent to healthcare staff, time to alert acknowledgement and whether patients with deterioration were identified and treated. The pilot study hopes to assess the use of wearable sensors and digital alerting to healthcare staff in an allocated hospital ward prior to more widespread rollout across the hospital and larger research trials.

## Methods

### Study design

A pilot study was performed on the use of wearable sensors and digital alerts sent to members of the healthcare team in real time. This is a prospective study with sensor data and ward observations taken in real time.

### Ethics

Ethical approval was granted by Yorkshire & The Humber - Leeds East Research Ethics Committee (reference number 17/YH/0296).

### Sensium sensor

The Sensium wearable sensor (The Surgical Company, Abingdon, UK) measures the vital signs: heart rate (HR), respiratory rate (RR) and temperature. It is lightweight and transmits data wirelessly via low-power radio frequency to engineered bridges which further transmit the data to a server. The data flows from the sensor via a bridge to the virtual server before it is sent via Wi-Fi to mobile applications on smartphone devices. All sensors are single use and have a battery life for 5 days, and patients requiring a further hospital stay will require another sensor to be placed.

A plastic strip is pulled to activate the sensor. The sensor records in a sequential cyclical 2-min fashion. A predictive strategy is used to calculate HR based on the R-R interval [23]. This approach has been described previously [24, 25]. The individual R-R intervals from the ECG strip are rank ordered and the median value is taken as the average HR. Impedance pneumography [IP] is the technique used to measure RR by the Sensium sensor. This is a common technique used to measure a person’s breathing rate [26]. IP is measured through superficial electrodes. The impedance measures both the respiratory volume and rate via the relationship between depth and thoracic impedance change [27]. The RR is derived from changes in the impedance of the thorax due to inhalation and exhalation. A very small current (iK) is injected through the ECG electrodes. The thoracic impedance changes are detected as variations in the voltage (V) measured at the ECG electrodes. Inhalation (peak resistance) and exhalation (trough resistance) are detected from a 60-s segment of IP waveform to calculate a median RR. Temperature is measured using a calibrated thermistor which is placed in the patient’s axilla and is a temperature-sensitive resistor. Individual vital sign readings are measured and processed in time order.

Once the physiological trace has been captured, there is an algorithm within the microchip found on the sensor. This has an inbuilt processing unit which transmits the average values of HR as beats per minute and RR as breaths per minute, to the nearest bridge. It is then transmitted to the central server [23] allowing digital alerts to be sent to healthcare staff through smartphones or electronic health records.

**Fig. 1:**
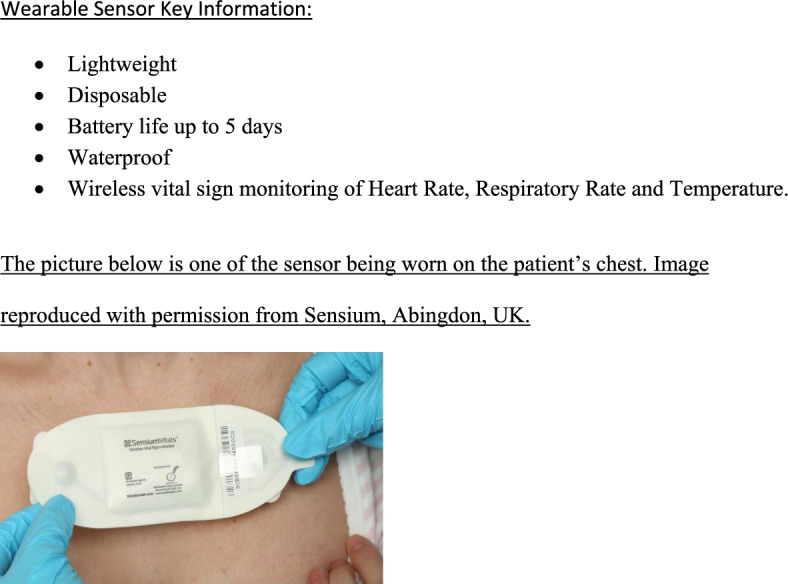
Properties of the wearable sensor

Figure 1 shows the properties of the wearable sensor. The sensor was either placed by trained healthcare professionals looking after the patient or the research team. The sensor was attached to the anterior chest wall using two standard disposable ECG electrodes (Red-Dot2560 3M, St Paul Minn). The medical tape was used to ensure that the temperature probe was secured in the axilla. Pre-processing of the data occurred to discard signals that were subject to gross electrical and motion artefact [28].

### Study location and participants

The surgical assessment unit (SAU) was chosen for recruitment as this is where new acutely unwell surgical patients are admitted.

Inclusion criteria:

- Consecutive patients above 18 years old with the capacity to give informed consent and who were predicted by clinical teams to remain in the hospital for 24 h or more were eligible for recruitment. This was based on the best clinical judgement of the lead treating physician. Only the senior most member of the team was consulted; in the UK, this is the consultant surgeon or medical physician. Those patients requiring a simple intervention such as a drainage of an abscess were not included whereas those patients likely to stay at least 24 h based on their clinical condition were included. An information sheet outlaying the sensor technology was given to all eligible patients. A verbal briefing was given by a member of the research team in addition to the written material. Written consent was obtained for all patients taking part in the study.

Exclusion criteria included:

- If any patient was unable to consent or withdrew their consent
- Patients with a skin condition on the chest wall
- Patients with an open chest wound
- Patients with mental health problems. The mental health assessment was judged through a combination of the assessment made by the senior attending clinician and the past medical history of the patient
- Patients with a pacemaker or defibrillator
- Any condition in which the judgement of the clinical team excluded patching

To our knowledge, this is one of the first studies on real-time digital alerting through wearable sensors and therefore data will be used to generate sample size estimates for later studies. A sample size of 50 was selected to be sufficient to draw conclusions and amend the study design for future iterations [24].

### Recruitment

The pilot study was commenced in May 2019 and was completed in August 2019. This was a prospective study with sequential recruitment and no randomisation.

### Staff training

A copy of all staff rotas for the nurses was obtained and staff training was performed between April and May 2019. The training and support were provided by Sensium. Nurses were taken from their clinical duties for an afternoon. Small group training (with 2–3 nurses) was performed with a trained nurse from the company. Training consisted of an online training session and a hands-on training session in which staff were required to place the sensor on a participant and enter the patient onto the Sensium system. A short online assessment was performed by all participants at the end of training to test appropriate levels of knowledge and understanding prior to sensor use. Only those staff members with the training that has passed the final assessment were able to place the sensor and monitor patients with it. The company was available daily during the trial for any questions that arose.

### The alerting device

The mobile application required several key actions to be recorded:

1. The exact date and time stamp when the patient had deteriorated
2. The time in which a member of staff acknowledged patient deterioration based on vital signs from the sensor
3. The times for when the clinical team identified a patient with potential infection
4. The times for when the clinical team identified a patient with sepsis

A list of actions on the application to be acknowledged by nurses when changes in vital signs were detected can be found in Table 1.

**Table 1:**
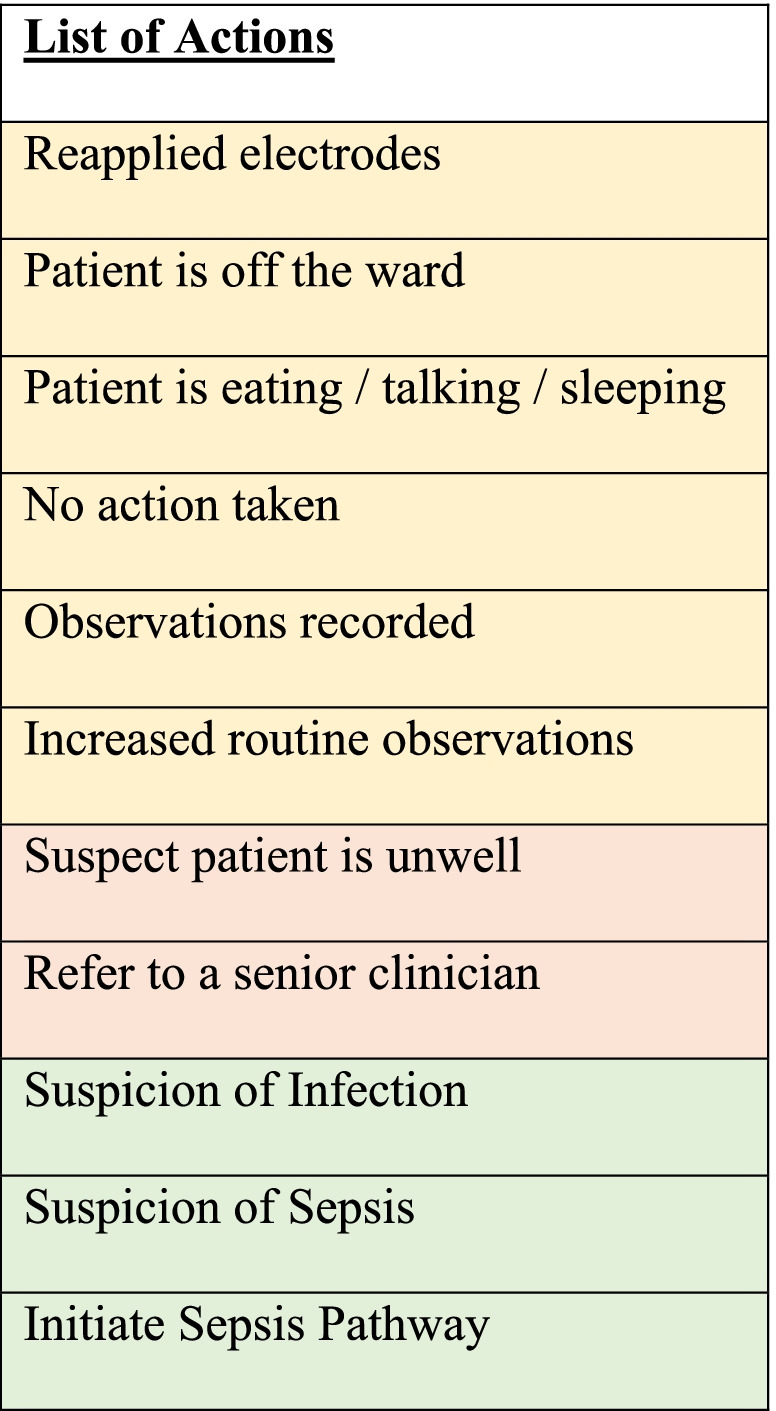
List of actions on the application to be acknowledged by nurses when there are changes in vital signs

**Fig. 2:**
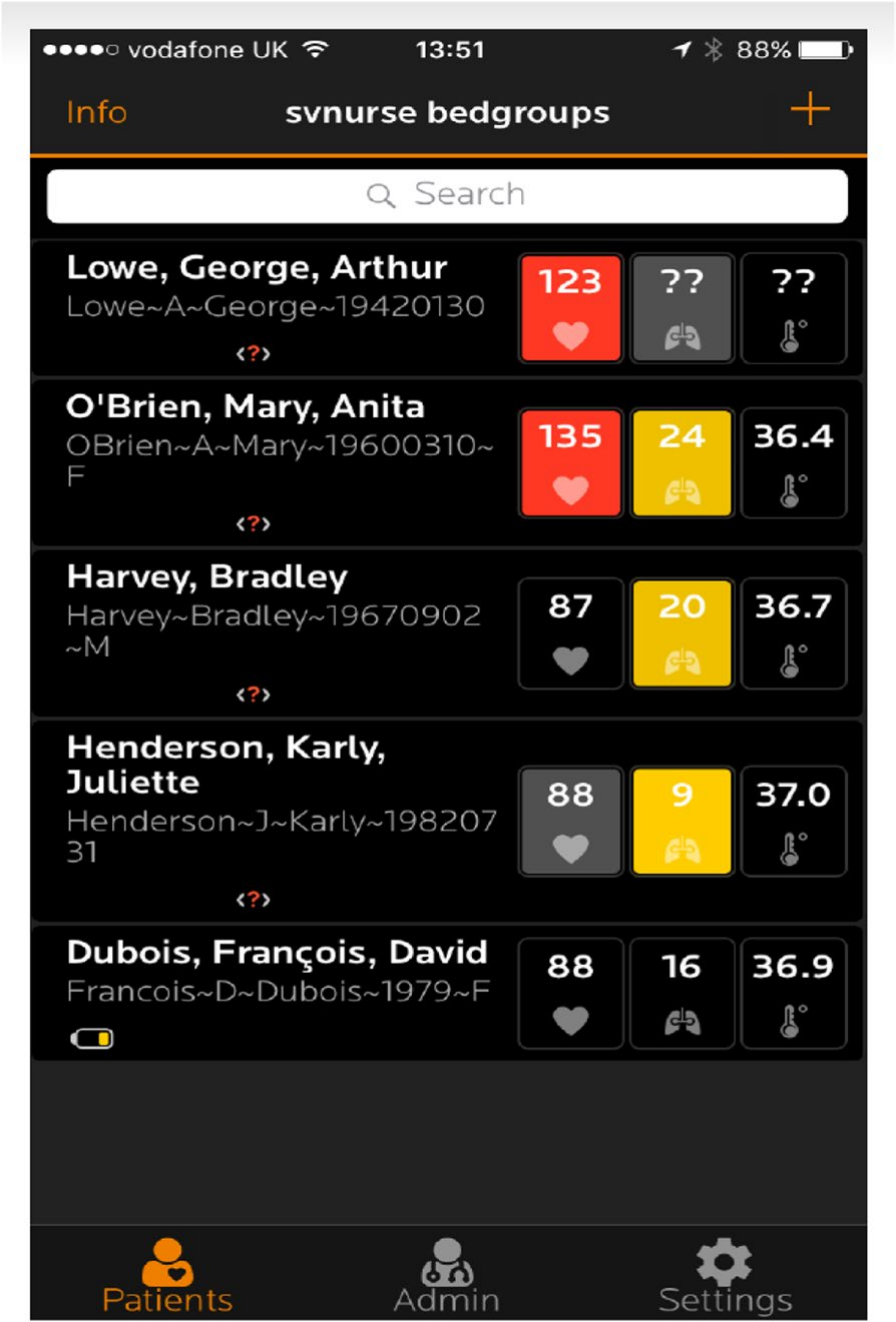
An image of the home screen with patients highlighted. All names, bed numbers and vital signs shown in the display are for demonstration purposes and not real patient data. The Sensium® App. Image used with permission from SENSIUM (Abingdon, UK)

The nurses on the ward looking after the patient were the same nurses that took routine observations and managed electronic alerts on the device. This ensured there was no bias between nurses taking manual observations and reviewing alerting on the sensor. Each nurse was given a smartphone at the start of the shift and this was left to charge at the end of the shift. All trained members of staff were given a secure username and password to log onto the application on the smartphone. As well as the handheld devices, there was also one central desktop on a large screen with the application downloaded and only used for the study if required. The research team and the Sensium nurse champion were all available to support the healthcare team with the application and the sensor. A ‘champion’ was a member of staff that was available on shift as a point of contact if any assistance was required. The ‘champions’ assisted with sensor placement and monitoring as well as offering assistance for other team members. In addition, there is an online help section for all users of the application, which guides users on the functionality of the application. End-users typically interfaced with a home screen which showed all the patients within a certain bed group (Fig. 2).

### Study procedure

If there was a vital sign that was outside the normal range for 10 min, an alert was generated, and the vital sign would flash on the display screen. This sign would only stop flashing if the alert had been acknowledged. If a patient deteriorated on the ward, an alert was sent to the designated nurse looking after that patient. When an alert is acknowledged, it is time stamped. The staff acknowledgement in the study is the time taken to acknowledge an alert on the handheld device. The nurses have been told that all alerts must be acknowledged in a timely manner. If the alert was not acknowledged, a reminder notification was sent to the nurse again 15 min later. Alerting continued until it was acknowledged by a member of the healthcare team. If there was no alert acknowledgement within 30 min, an alert was sent to the senior nurse in charge of the ward. For the purpose of this study, the alerts were only sent to nurses on the ward.

Upon patient discharge, all ward observations were collected and transcribed onto Excel sheets to allow for further comparisons with sensor data. The discharge summary and clinical notes were reviewed for any significant events of patient deterioration and the clinical course of events experienced by the patient whilst in the hospital. Clerking notes were reviewed for any signs of sepsis, severe infection or suspicion of infection. The diagnosis of sepsis was also checked with the senior doctor in charge of the patient whilst in hospital (in the UK, this is typically the consultant). All alerts were reviewed for recruited patients to identify the exact time on the sensor that deterioration had occurred. The average time to acknowledgement was calculated. The pilot study hopes to assess the use of wearable sensors and digital alerting to healthcare staff in an allocated hospital ward prior to more widespread rollout across the hospital and larger research trials.

## Results

### Total number of vital sign alert notifications

There were 50 patients recruited in this pilot study, of which there were vital sign alerts in 18 patients (36%). The total number of vital sign alerts generated in these 18 patients was 51. Of these 51 alerts, there were 7 alerts for high HR (13.7%), 33 for RR (64.7%) and 11 for temperature (21.6%).

For the 51 alerts generated, there were 27 alerts that were acknowledged, 23 notifications that were left unacknowledged and one alert with no sensor or ward timestamp. In those left unacknowledged, there were 4 alerts for HR, 16 for RR and 3 for temperature.

Out of the 27 acknowledged alerts, there were 2 alerts for HR, 17 for RR and 8 for temperature.

The average time to staff acknowledgement of the notification for all alerts was 154 min (2.6 h).

The average time to staff acknowledgement for HR was 179 min (2.98 h).

The average time to staff acknowledgement for RR was 162 min (2.7 h).

The average time to staff acknowledgement for temperature was 165 min (2.75 h).

All notifications sent, acknowledgements and timestamps can be found in Table 2.

**Table 2:**
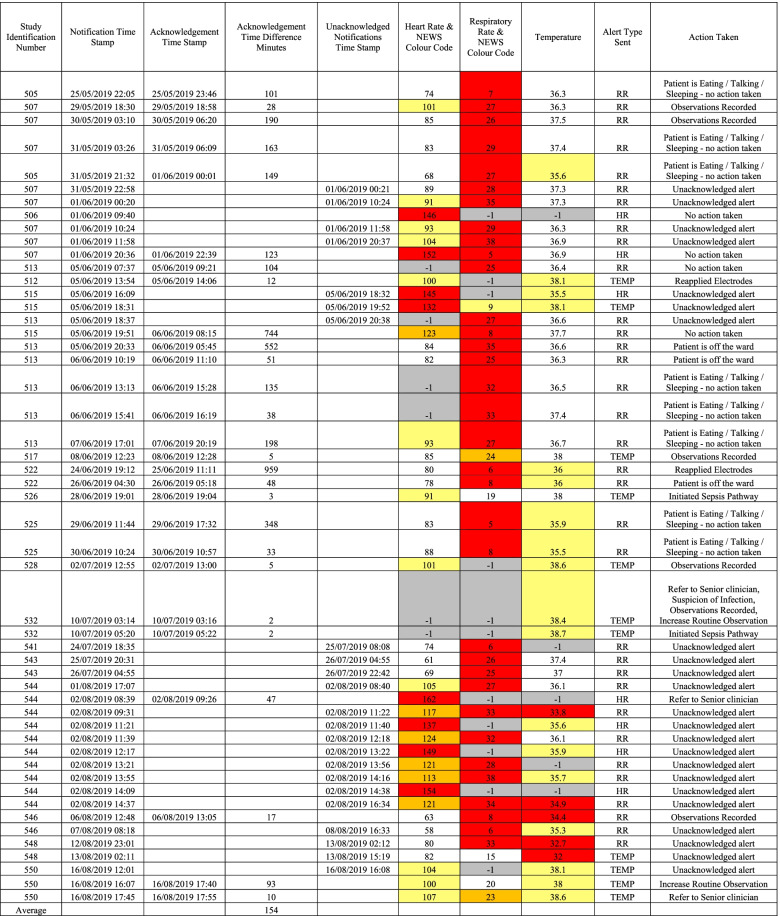
Sensor vital sign notifications, acknowledgements and actions taken by the clinical team

### Alert actions taken

The most frequent acknowledgement by nursing staff was to say that the patient was either eating, talking or sleeping (8 acknowledgements, 29.6% of all acknowledgements). The alerts prompted nurses to record a full set of manual observations in 6 cases. There was no action taken in 4 cases. Some acknowledgements were to say the patient was off the ward (3 cases) and sensor electrodes were replaced 2 cases. There was one patient with no alert time either by the sensor or the ward observations.

A total of 18 patients had shown signs of deterioration in the cohort based on vital sign changes on the sensor. The frequency of routine observation monitoring was increased in 2 cases, 3 patients were referred to a senior clinician and 2 patients were initiated on the sepsis pathway.

## Discussion

This small pilot study used wearable sensors to generate real-time alerts to nursing staff when changes in vital signs were found. This paper demonstrates the clinical implementation of wearable sensors and offers practical clinical value. Research has highlighted that deterioration in vital signs especially HR and RR precede adverse events providing an opportunity to act on patients earlier, helping to provide treatment and even prevent more serious events from occurring [25–27]. The sensor provides continuous monitoring enabling earlier detection of patient deterioration. It is hoped that through this earlier detection the worsening spiral of deterioration that patients undergo may be halted. In intermittent monitoring, this opportunity may be lost.

Wearable sensors are not routinely used to measure vital signs within the NHS or in a wider setting. Research on the successful implementation of wearable sensors in vital sign monitoring in the literature is limited with small numbers of patients [19, 29]. In addition, the focus of these papers has been on sensor reliability with no review of time to clinical team acknowledgement [19, 29]. Across the world, there is a shortage of trained nursing staff and this has been associated with adverse outcomes such as missing patient deterioration and patient mortality [30]. Whilst the new technology is not in any way being used as a replacement for nursing staff, it may be used to help bridge the gap and guide staff appropriately to deteriorating patients. The wearable sensor would be used in addition to more classical monitoring. It is hoped that the two would act synergistically with the sensor providing continuous monitoring and classical monitoring permitting face-to-face contact and rapport as well as an opportunity for clinical patient assessment. Whilst wearable sensors can measure physiological parameters, other important assessments such as cognitive function, mood and pain levels are not assessed.

This paper has shown that although digital alerts were sent to the nursing staff they were not always acknowledged and if they were acknowledged this was not done in a timely manner.

There are several reasons that the delay in acknowledgement or failure to acknowledge the alerts may have occurred. The first possibility is that the alert prompted a member of staff to review the patient but there was a failure to click the acknowledgement. The second possibility is through a lack of education and the clinical team being unaware that clicking the acknowledgement was important. The third possibility more worryingly is that the patient may not have been reviewed. Unfortunately, the only way that would have been known is if there were observers throughout the entire study period which was not possible.

The final possibility of alerts not being acknowledged is through alarm fatigue [31–33]. Alarm fatigue has been thoroughly documented in the literature and may help to explain some of the findings. Much of the work in alarm fatigue is based in critical care units due to high numbers of false alarms and non-actionable (true but clinically irrelevant) alarms [34]. In alarm fatigue, the clinical teams may ignore or silence alarms missing critical changes in the patient’s condition [35, 36]. There are new strategies being developed to help reduce the risk of alarm fatigue such as clinical decision software [34]. The software is being increasingly used to provide an alert on individual patient data and being tested within the intensive care setting [34]. Although alarm fatigue has been suggested, it is unlikely that this is the main reason to explain the findings. In the intensive care setting, all patients have increased monitoring; however, on the ward at any one time in the study, there was a maximum of five patients wearing the sensor. The number of alarms generated from these five patients was likely to be minimal.

It has been shown in the literature that some alerts are more effective than others but it is unknown why. In a systematic review, the majority of digital alerts improved outcome; however, there are some cases where the outcomes did not improve [33]. Alert management programmes must ultimately meet the goal of both improving patient care and at the same time reducing the alert burden on clinicians [32]. Clinicians are less likely to accept alerts when a greater number of alerts are received, especially if the alerts are repeated [31]. Reducing within-patient alert repeats may be a promising way of reducing alert fatigue [31].

Despite the failure to acknowledge some alerts, in some cases, changes in vital signs were acknowledged. In cases of deterioration or potential sepsis, all alerts were acknowledged. In two patients, the sepsis pathway was started, and in three patients, there was a referral made to a senior clinician. It is unknown whether the sepsis pathway was started due to the alert or if it may have occurred without it; however, the alerts will have acted as a prompt to review the patient and act accordingly.

This paper has several key strengths and shows the implementation of wearable sensors and real-time alerting in acutely unwell surgical patients. This paper for the first time reviews real-time alert generation from wearable sensor technology and time to alert acknowledgement. All members of regular nursing staff within the clinical area received training and completed an assessment prior to the study starting. The training of staff members took several weeks; however, it was very important if widespread use was to take place on the ward. All clinical areas were successfully engineered for use.

This paper has several limitations which must be considered. The pilot nature of this study means that a larger trial is required with a greater number of patients to review full-scale deployment of wearable sensors and review outcomes. Outcomes may include the identification of patient deterioration, referrals to higher levels of care and whether there is a difference in patient length of stay. This is a single-centre study and the wearable sensors were only used when patients were in one clinical area (SAU). Although changes in vital signs were detected using the wearable sensors, it is hard to say that they would have remained undetected with manual ward observations. Unfortunately, 23 of 51 (45%) of sensor notifications were left unacknowledged. In these cases, it is unknown if nursing staff went on to review the patient and failed to acknowledge on the Sensium application or if they failed to register the change in vital sign at all and did not review the patient.

One of the challenges with the study which may help to explain some of the results is that nurses were reviewing the digital alerts from the sensor in addition to their current monitoring. They were using paper-based observation charts as well as having to review digital monitoring through smartphone technology. It is likely that this may have increased their nursing workload. If nurses are reviewing digital alerts in addition to their ward monitoring, they may have felt this was an extra job in addition to their already busy workload. Ideally, the nurses would have already been using digital vital sign monitoring with alerts from wearable sensors fully integrated into such systems. Once this occurs, alerts from wearable sensors may be incorporated into a more standard operating procedure rather than being an additional task for nurses to review. The pilot nature of the design means conclusions should be drawn with caution. Environmental factors may have played a role and further studies are required to decipher if the study findings are consistent across other hospital settings.

### Further research

This paper highlights that although wearable sensor technology allows more continuous monitoring the alerts generated to the nursing team were not always acknowledged. Further research is required to help better understand why this occurs. In the patients with alert acknowledgement, there was often a delay. Reasons as to why these delays are still occurring must be explored in further research. A larger study will be required with more than one clinical area used, larger rollout of wearable sensors across the hospital will help. Further research could entail repeating the study; however, this time having trained observers available to record why the nurses were acknowledging some alerts and not others. In addition, qualitative research conducted with nurses may answer some of these questions. Even in the small cohort of patients recruited, 2 patients with sepsis were identified and started on the sepsis pathway and 3 patients were referred to a senior clinician.

The use of paper observation charts in addition to wearable sensor digital alerts may have increased the workload and demands on nurses. More fully integrated systems may enable notifications to be acted upon and acknowledged in a timely manner.

As well as integration, the other key to review in future is the effect of digital alerting through wearable sensors and patient outcomes. One such study in the literature found continuous monitoring on ward patients through sensor technology resulted in a reduction in hospital and intensive care length of stay as well as lower rates of ‘code blue’ or cardiac arrest [37]. This study has been welcomed and was the first to review outcomes [37]. However, there are several significant limitations found. Firstly, the sensor being used was not quite a wearable sensor, but the Early Sense sensor which is a sensor is placed under the patient’s mattress and only records whilst the patient is in bed. For ambulating ward patients, its use and applicability may be limited. In addition, only length of stay and code blue were reviewed; other outcome measures of interest such as mortality, transfers to intensive care and rapid response teams were unable to be reviewed. In another more recent study, an improvement in patient outcomes was found with significant cost savings; however, the authors concluded that further research is required to review wearable sensors and all outcome measures in a larger cohort of patients [38].

## Conclusion

This paper has seen the implementation of digital alerts based on developed alerting algorithms to nurses in real time. Although not all alerts were acknowledged, deterioration on the ward observations was detected and actions were taken accordingly. Patients were started on the sepsis pathway and escalation to senior clinicians occurred. Full integration of paper-based ward observations and digital alerting from wearable sensors using electronic health systems needs to be explored and is likely to improve results.

## Data Availability

The datasets during and/or analysed during the current study are available from the corresponding author on reasonable request.
